# The relationship between business competition and welfare in Indonesia

**DOI:** 10.1371/journal.pone.0308877

**Published:** 2025-01-21

**Authors:** Maman Setiawan, Rina Indiastuti, Nury Effendi

**Affiliations:** Department of Economics, Faculty of Economics and Business, Universitas Padjadjaran, Bandung, Jawa Barat, Indonesia; PLoS ONE, UNITED STATES OF AMERICA

## Abstract

This research freshly investigates the effect of business competition on national welfare in Indonesia. This research uses business competition index data obtained from the Indonesia Competition Commission (KPPU) and welfare indicators data collected from the Indonesian Bureau of Central Statistics (BPS) at the economic sectoral and provincial levels for the period 2018–2020. The relationship between business competition and welfare is estimated using pooled regression model combining year, economic sector, and province. This research also uses business competition index applying both same weights for all dimensions of competition and the weights derived from principal component analysis. This research finds that business competition has a positive effect on welfare as represented by gross regional domestic product, regional productivity, productivity growth, wages, and human development index. Furthermore, the regions with high scores on the competition index mostly come from Java Island. Therefore, Indonesian government must encourage the mainstreaming of business competition in all provinces in Indonesia.

## 1. Introduction

The practice of fair business competition is positively correlated with national welfare [1, 2]. The national welfare affected by the competition can be widely defined, not only including gross domestic product (GDP) and productivity, but also employment, wages, and human development index (see [1–4]). The impact of competition on national welfare can be seen through several channels, including innovation, efficient use of resources, and labor market performance [2]. Because to the intense competition, businesses are under pressure to innovate and use resources more efficient in order to boost productivity and maintain competitive product pricing, which may lead to a rise in the demand for labor and higher real wages ([5, 6] argued that competition was only good for value capturing instead of value creation. Therefore, the positive effect of competition on the welfare may also come from the value capturing effect). Thus, the efficiency effect of competition can encourage welfare (see [7]).

Previous studies have investigated the impact of competition on several indicators of welfare including productivity, efficiency, and economic growth. However, research about the effects of competition on efficiency and productivity is still limited to several sectors, such as the manufacturing and banking industries. Furthermore, research about the impact of competition on national welfare is also rare where previous research only investigated the effect of competition policy on economic growth and also did not apply business competition measure and other indicators of national welfare (see [8]). In addition, [9] investigated the impact of competition on welfare theoretically in an anonymous two–sided matching market using game theory. The later did not provide empirical evidence. Regarding that, there is still contribution of empirical research in investigating the competition on national welfare using a business or national competition measure and various national welfare indicators.

Furthermore, the impact of business competition on welfare in Indonesia was also previously investigated for specific Indonesian micro sectors. For example, [10–13] only investigated the impact of market structure on firm price-cost margins in the Indonesian food and beverage industry. Moreover, [14] investigated the relationship between industrial concentration and price-cost margin in the Indonesian manufacturing industry. [15] also investigated the effect of industrial concentration on cost efficiency in the Indonesian banking sector. Thus, research about the effect of business competition on welfare using the national context in Indonesia is still important.

Moreover, the estimation of competition measures is still limited to the partial measures of market structure and performance, such as industry concentration and mark-up, and the relationship between these variables [10–13, 16–18]. Applying the partial measure of competition and measuring the competition only for few sectors might not represent the real national competition. Also, recent research by [19] applied structure, conduct and performance to measure the competition, but this still neglected the environmental factors forming the competition measure (see [20]). Therefore, it is important to investigate the impact of business competition on national welfare using more comprehensive measures in Indonesia.

Regarding business competition and national welfare in Indonesia, Table 1 indicates the trend of the business competition index and national welfare, represented by gross domestic product, during period 2018–2020. From Table 1 it can be shown that there was an indication of a positive trend between competition index and GDP. Competition may affect the GDP and other indicators of welfare by improving the efficiency and productivity. Thus, it is relevant to empirically investigate the relationship between competition and national welfare in Indonesia.

**Table 1:** Competition index and gross domestic product.

Year	Competition Index	GDP (Billion Rupiah)
		(2010 = 100)
2018	4.63	10,425,851.9
2019	4.72	10,949,155.4
2020	4.65	10,722,999.3

Sources: BPS and Indonesia Competition Commission (KPPU)

Based on the previous background, this research investigates the relation between business competition and welfare in Indonesia. This research applied data from Bureau of Central Statistics (https://www.bps.go.id/id/publication) for the welfare measures. The competition measure was represented by business competition index sourced from Indonesian Competition Commission. To investigate the relation between competition and welfare, this research uses pooled regression model combining data from economic sectors, provinces and years. This research found that the regions that had high welfare tend to have a high level of business competition. This research generally found a positive relationship between the business competition index and national welfare.

This research has two contributions i.e. (i) this research provides a comprehensive national business competition which is hardly found in the previous literature and (ii) this research estimates the relationship between business competition and national welfare, which is still rare, especially in Indonesia. The paper is organized as follows: Section 2 presents a review of the literature; Section 3 describes the method and the data; and Section 4 presents the model estimation and results. The final section summarizes the findings and presents the research’s conclusions and policy implications.

## 2. Literature review

The impact of business competition on welfare has been investigated using a number of different measures (The discussion of welfare measure was also discussed in [4]). [20, 21] used a mathematical formulation to examine the relationships between business competition and economic growth. The formulation indicated that there was a positive impact of competition on market expansion through advanced technology and innovation. Furthermore, [8] used the traditional Solow growth model to examine the impact of firm competition, as measured by competition regulation, on economic growth in 101 countries. The findings indicate that competition regulation is ineffective at increasing economic growth. The research of [22] also revealed that gross domestic product per capita growth is related to the level of regulatory stringency as a measure of competition. Most of the previous research applied competition regulation as a measure of competition affecting the economic growth, since there was a lack of national competition measure.

Regarding the impact of business competition on productivity, [18, 23, 24] showed in their findings that high levels of firm productivity were correlated with low industrial concentration. Also, [25] found that economic competition can increase productivity. Moreover, the research by [26] showed that competition increases employee productivity. Supporting the productivity effect of competition, [9] theoretically investigated the relationship between competition and market efficiency in an anonymous two–sided matching market using game theory. The research found that competition might increase the market efficiency which may naturally affect the productivity. Also, [27] found that competition increased productivity growth in Australia.

Regarding the relationship between the job market (as another measure of national welfare) and business competition, [28] found that a market with high competition will promote job creation. Furthermore, [29] developed a model framework to examine the relationships between business competition, job creation, and economic growth in European nations. According to the findings of their study, competitive pressures that can foster competition and are pursued thoroughly can support input demand, especially labor demand, and increase economic growth. Additionally, [30] carried out a comparable study in the UK and discovered that there were better labor partnerships following increased business competition. On the other hand, [31] found that an increase in market concentration could reduce the level of total employment. This is supported by [32], who examined the effect of competition on job security in Spain. The latter concluded that the higher the business competition, the lower the job security. The recent research from [33] also found that the lower competition represented by higher market power decreased employment.

Furthermore, several previous studies used wages as an indicator of national welfare, which was influenced by business competition. According to [34], an increase in the number of firms entering the market caused the firm’s market share to be lower. It caused more intense competition and could increase wage levels. This study’s results align with [35], whose research explained that wages in concentrated industries were lower than wages in non-concentrated industries [36]. In addition, business competition can increase real wages through a decrease in product prices, so that the purchasing power of wages increases relative to product prices. Through an increase in real wages, the structural unemployment rate will be lower because there will be no incentive for workers to change jobs or leave their jobs [37–39].

According to previous research, indicators of national welfare can also be reflected in economic and non-economic aspects, such as the human development index (HDI), through improving health and education. [40] showed a positive relationship between hospital competition and health care improvements in China using individual and province-level data. In addition, [41] found that an increase in the number of hospitals in the United States could increase competition and benefit consumers. Meanwhile, [42] concluded that competition in the higher education market increased the quality of higher education services in New Zealand. Also, [43] found that higher education competition increased global competition and the quality of education. Therefore, it can be concluded that increasing competition in the health and education sectors can increase the HDI.

Based on the previous research, it is hardly found research investigating the impact of business competition on welfare in Indonesia. Also, previous research did not apply a real business competition measure at regional and sectoral levels. Thus, this research has an academic contribution by providing a measure of the business competition index at regional and sectoral levels as well as using comprehensive measures of welfare.

## 3. Methodology and data

### 3.1. Research model

This research uses modified models from previous studies by [8, 22, 44, 45]. Previous research models used a national production approach, which directly connected business competition with economic welfare as measured by productivity and its growth. The first model of the impact of business competition on welfare follows the modified models of [22, 44, 45] by introducing the impact of Covid-19 Pandemic (The impact of Covid-19 pandemic on the national welfare can be seen in [46].), as follows:

$$Ln{\left[\frac{Y}{L}\right]}_{it}=\alpha_{0}+\alpha_{l}\ln{\left(S_{k}\right)}_{it}+\alpha_{2}\ln{\left(S_{h}\right)}_{it}+\alpha_{3}\ln{\left(n+g+\delta \right)}_{it}+\alpha_{4}\ln{\left(\phi \right)}_{it}+\alpha_{5}\mathrm{Dcov}+\sum \delta_{i}D\mathrm{cov}*X_{it}+\varepsilon_{it}$$ (1)

The second model follows the research of Ma (2011) by having the modified model, as follows:

$${\mathrm{Growth}}_{it}=\eta_{0}+\eta_{1}\ln{\left(S_{k}\right)}_{it}+\eta_{2}\ln{\left(n+g+\delta \right)}_{it}+\eta_{3}\ln{\left(Y/L\right)}_{it}+\eta_{4}\ln{\left(\phi \right)}_{it}+\eta_{5}D\mathrm{cov}+\sum \phi_{i}D\mathrm{cov}*R_{it}+\epsilon_{it}$$ (2)

where *i* and *t* index consecutive regional and year; Y is regional gross domestic product at province level (GRDP) (This research uses GRDP because the business competition index provided by KPPU is presented at the provincial level. Moreover, using total of national GDP cannot be applied because of the short period of 2018–2020.); Y/L is the ratio between regional gross domestic product and labor (regional productivity); *ϕ* is the business competition; *s*_*k*_ is the ratio between investment to gross regional domestic product; *s*_*h*_ is the ratio between human resource expenditures to regional gross domestic product; *g* is population growth; *Growth* is Y/L growth; X is the vector of variables including *s*_*h*,_ *s*_*k*,_ *and ϕ*; R is the vector of variables including *s*_*k*_ *and ϕ*; Dcov is year dummy of Covid-19 pandemic in 2020.

The third model follows previous research using other welfare measures that are affected by the competition such as employment [28–32], wage [34–39] and HDI [40–43]. Other control variables (vector of W) are also taken from the models of [8, 22, 44, 45] based on the econometrics modelling to obtain unbiased parameters in the model, as follows:

$$\ln Z_{it}=\beta_{0}+\beta_{1}\ln{\left(\phi \right)}_{it}+\sum \theta_{i}W_{it}+\sum \mu_{i}D\mathrm{cov}*W_{it}+\varepsilon_{it}$$ (3)

where Z are other indicators of welfare (labor, wage, and human development index).

Before estimating the models (1)-(3), this research also estimates a single regression model associating business competition with gross regional domestic product to give a direction in estimating the effect of competition on welfare, as follows:

$$lnY_{it}={£}_{0}+{£}_{1}ln{\left(\phi \right)}_{it}+{€}_{it}$$ (4)

In addition, this research applies instrumental variables for the (Y/L) variable in the Eq (2), including domestic investment and foreign investment, year dummy variables (2018–2020 period), and regional dummy variables (34 provinces) (This research finds the endogeneity problem in the variable of Y/L in the Eq (2)).

### 3.2. Data

This research uses data or research variables sourced from the Commission of the Supervision of Business Competition (KPPU) and Indonesian Bureau of Central Statistics (BPS) reports. The research period uses data from the 2018–2020 period, adjusted for the availability of business competition index data, which will be used as a measure of business competition in this study. Business competition index data is obtained from KPPU, which can be derived at the economic and/or provincial sectoral level. Regarding national welfare measures, this research uses sectoral economic and/or provincial data sourced from BPS that were observed during the 2018–2020 period. Welfare measures to be used include gross regional domestic product, regional output productivity and its growth, labor, wage, and the human development index.

This research applies the business competition index surveyed by KPPU to measure business competition. The business competition index surveyed by Indonesia Competition Commission (KPPU) has 7 (seven) dimensions including structure, conduct, performance, institution, regulation, demand and supply [47–49]. Each dimension has relevant indicators that are related to the competition measures. The business competition index was resulted from a survey of 34 provinces in Indonesia. The respondents for the survey included Central Bank of Indonesia; Indonesian Chambers of Commerce and Industry; trade and industry agency; and academics. Regarding the business competition index, this research applies two data of business competition index provided by KPPU i.e. (i) business competition index with the same weight for all dimensions of competition and (ii) business competition index with the weights derived from principal component analysis (PCA). In spite of this, KPPU reported only the business competition index using the same weight as the final indicator of the national business competition. The business competition index using the weights from PCA is also applied to check the robustness of the index. The business competition index has a score in the range between 1 and 7.

Regarding the variables applied in the Eq (1) until Eq (4), this research uses data to measure the variables based on the previous research. The gross domestic regional product of each sector in every province was sourced from Bureau of Central Statistics (BPS) using 2010 constant market price. Labor was measured by number of employed workers or working population sourced from The National Labor Force Survey provided by BPS. Investment was measured using the total domestic and foreign investment sourced from BPS and Indonesian Coordinating Investment Board. Human resource expenditure was represented by the expenditures for employment sourced from BPS. The wage was measured by the wage per worker sourced from BPS. The data of human development index for every province was already surveyed and provided by BPS. Dummy of Covid-19 pandemic in 2020 was assumed the value 1 for the year 2020 when the Covid-19 pandemic began and 0 otherwise.

### 3.3. Estimation strategy

This research analyses the impact of business competition on national welfare. Due to data limitations, the estimates made are as follows:

1. The relationship between GRDP and business competition in a single regression model is estimated using pooled data combining year, economic sector, and province.
2. The relationship between GRDP and business competition is also carried out in a single regression model using pooled data combining only data of provinces and years and combining only data of economic sectors and years as a comparison.
3. Models (1)-(3), which analyse the relationship between business competition and welfare and other determinants, are estimated using pooled data combining province and year because estimation at the annual sectoral level is impossible due to the unavailability of data at the sectoral level for the control variables.

## 4. Results

### 4.1. Description of business competition conditions

Tables 2–4 show business competition index variables, grouped by economic sector and province, that were used to examine the level of national business competition (The business competition index presented in the Tables 2 and 3 are calculated using the same weights for all dimensions). The sectors with the highest business competition index (≥5) during period 2018–2020 were wholesale and retail trade, auto and motorcycle repair; and food and beverage providers. The top six economic sectors with the highest business competition index also included the following four sectors: financial and insurance services; educational services; information and communication services; and business services. Economic sectors with a high competition index were not always sectors that contributed significantly to the national economy.

**Table 2:** Average sectoral GRDP and sectoral business competition index, 2018–2020.

Sector	GRDP	Competition Index
Agriculture, Forestry, and Fisheries	55465.33	4.69
Mining and excavation	29903.00	4.40
Processing industry	6276.33	4.71
Procurement of Electricity, Gas	1095.33	4.19
Water Supply, Waste Management, Waste and Recycling	361.67	4.24
Construction	18251.00	4.69
Wholesale and Retail Trade, Car and Motorcycle Repair	17305.67	5.01
Transportation and Warehousing	4477.67	4.78
Provision of Accommodation and Food and Drink	13140.00	5.00
Information and Communication	31494.67	4.74
Financial Services and Insurance	1785.00	4.84
Real Estate	9661.33	4.78
Company Services	664.00	4.74
Education Services	3461.67	4.80
Health Services and Social Activities	1107.33	4.77

Source: authors’ calculation

**Table 3:** Average GRDP and regional business competition index by province, 2018–2020.

Province	GRDP	Competition Index	Province	GRDP	Competition Index
Aceh	130161	4.49	West Nusa Tenggara	92496	5.09
North Sumatra	528674.3	4.75	East Nusa Tenggara	68040.66	4.56
West Sumatra	168556	4.59	West Kalimantan	134194	4.89
Riau	489229	4.88	Central Kalimantan	97960.34	4.51
Jambi	146831.7	4.43	South Kalimantan	130730.3	4.72
South Sumatra	309700.3	4.79	East Kalimantan	474757	4.65
Bengkulu	45615.67	4.45	North Kalimantan	59875	4.66
Lampung	238951	4.35	North Sulawesi	87128.34	4.62
Bangka Belitung Islands	52950	4.48	Central Sulawesi	126548	5.02
Riau islands	176790.7	4.88	South Sulawesi	322618.3	4.79
Jakarta	1788067	5.45	Southeast Sulawesi	91936.66	4.51
West Java	1455478	5.07	Gorontalo	27856.33	4.16
Central Java	966211	5.13	West Sulawesi	32024.67	4.23
Yogyakarta	101397.3	4.87	Maluku	30423.67	4.35
East Java	1607877	5.10	North Maluku	26487.67	4.44
Banten	443940	4.71	West Papua	61377	3.91
Bali	154772.3	4.80	Papua	143984	3.54

Source: authors’ calculation

**Table 4:** Regression of business competition on GRDP (Y) (GRDP and business competition are transformed into natural logarithms in Tables 4 and 5).

Independent Variable	Dependent Variable: LnY		
	Model 1	Model 2	Model 3
	Coefficient	Coefficient	Coefficient
Intercept	-1.244	3.782**	-14.293***
	-1.44	-1.591	-1.815
Ln(*ϕ*)	5.635***	5.389***	17.520***
	-0.493	-1.034	-1.218
R^2^	0.368	0.23	0.39
F-statistics	25.54***	27.19***	207.03***
N	1470	95	45

Source: authors’ calculation

Note: Standard errors in parentheses

Model 1 uses pooled data combining economic sectors, provincial, and year.

Model 2 uses pooled data combining year and province.

Model 3 uses pooled data combing year and economic sectors.

** Significance at the 5% level

*** Significance at the 1% level

Based on Table 2, the sectors that have had an average competition index below 4.5 for the last three years are the mining and quarrying sector; water supply, waste management and recycling; and electricity and gas supply. Those sectors consistently stayed in the same ranks during the period 2018–2020. In general, these three sectors were not yet widely provided by the private sector, so business competition was still low.

Table 3 show the average business competition index for each province for the 2018–2020 period. During this period, DKI Jakarta was the province that had the highest business competition index, with an average business competition index score of 5.45. The other five regions that had a business competition index above 5 were Central Java, East Java, West Java, West Nusa Tenggara, and North Sulawesi. In general, the provinces on the island of Java dominated the five provinces with the highest business competition index. Furthermore, provinces with a high level of business competition index were also the provinces with the largest economies in Indonesia. With a very large scale of economy, there will be quite a number of business actors or companies operating in the province, thereby encouraging business competition in these provinces (This could cause the market efficiency in the Java Island was better than outside Java Island, which was also found in the research of [50]).

Table 3 also shows that five provinces had the lowest average business competition index during the 2018–2020 period, namely West Papua, Papua, Gorontalo, West Sulawesi, and Lampung. The provinces of West Papua, Papua, and Gorontalo have always been included in the five provinces with the lowest business competition index during the 2018–2020 period. Regions that have low business competition are generally located outside Java. West Papua and Papua are the two regions that have the lowest business competition, thought to be due to the small number of business actors in almost all sectors there. It is acknowledged that other regions, such as Gorontalo, West Sulawesi, and Lampung, did not yet offer enough attractiveness for business actors to move there and operate more businesses, as a result of which there is currently less business competition in these regions.

### 4.2. Business competition and welfare model

Tables 4–10 show the results of the effect of competition on welfare as formulated in Eq (1)–Eq (4). The results provided in Tables 4–10 have been gone through a battery of tests in econometrics modelling such as multicollinearity, heteroscedasticity and autocorrelation tests. The tests indicated that the results had no problems of multicollinearity, heteroscedasticity and autocorrelation tests (We applied the correlation test among the independent variables for the multicollinearity test. White and Durbin-Watson tests were applied to check whether there were problems of heteroscedasticity and autocorrelation, respectively).

**Table 5:** Regression of business competition (*ϕ)* on GRDP (Y).

Independent Variable	Dependent Variable: LnY		
	Model 1	Model 2	Model 3
	Coefficient	Coefficient	Coefficient
Intercept	-0.855	4.436***	-9.831***
	-0.838	-1.481	-0.318
Ln(*ϕ*)	5.446***	5.021***	14.648***
	-0.493	-0.973	-0.097
R^2^	0.369	0.222	0.352
F-statistics/Wald-Statistics	25.65***	26.64***	22772***
N	1470	95	45

Source: authors’ calculation

Note: Standard errors in parentheses

Model 1 uses panel data analysis units of economic sector, provincial, and annual data

Model 2 uses annual provincial units of analysis.

Model 3 uses annual economic sector units of analysis.

*** Significance at the 1% level

**Table 6:** Regression of business competition on productivity (Y/L).

Independent Variable	Dependent Variable: Ln(Y/L)	
	Model 1	Model 2
	Coefficient	Coefficient
Intercept	0.181*	0.176**
	(0.094)	(0.090)
Ln(*ϕ*)	0.010*	0.013***
	(0.006)	(0.004)
Ln(S_h_)	-0.021***	-0.022***
	(0.006)	(0.006)
Ln(S_k_)	-0.022**	-0.021**
	(0.009)	(0.009)
Ln*(n+g+δ)*^1^	-0.006	-0.006
	(0.009)	(0.009)
Dcov	-0.001	-0.0004
	(0.046)	(0.045)
Dcov* Ln(*ϕ*)	-0.013	-0.015***
	(0.008)	(0.006)
Dcov* Ln(S_h_)	-0.002	-0.002
	(0.003)	(0.003)
Dcov* Ln(S_k_)	0.003	0.003
	(0.006)	(0.006)
R^2^	0.57	0.58
Wald-statistics	63.71***	66.04***

Source: authors’ calculation

Note: Standard errors in parentheses

Model 1 uses business competition index with same weights of dimensions.

Model 2 uses business competition index with PCA weights of dimensions

* Significance at the 10% level

** Significance at the 5% level

*** Significance at the 1% level

^1^ Variable of *(n+g+δ)* is represented by growth of population as also applied by [8].

**Table 7:** Regression of business competition (*ϕ*) on productivity growth (Growth).

Independent Variable	Dependent Variable: Growth	
	Model 1	Model 2
	Coefficient	Coefficient
Intercept	-0.065	-0.098
	(0.133)	(0.129)
Ln(*ϕ*)	0.110	0.136**
	(0.080)	(0.069)
Ln(Y/L)	0.006	-0.003
	(0.043)	(0.043)
Ln(S_k_)	-0.015	-0.016
	(0.018)	(0.018)
Ln*(n+g+δ)*	-0.021***	-0.018**
	(0.007)	(0.008)
Dcov	-0.061	-0.059
	(0.239)	(0.226)
Dcov* Ln(*ϕ*)	-0.166	-0.174
	(0.145)	(0.132)
Dcov*Ln(Inv/Y)	0.049*	0.050*
	(0.029)	(0.029)
R^2^	0.24	0.27
Wald-statistics	94.75	90.83

Source: authors’ calculation

Note: Standard errors in parentheses

Model 1 uses the business competition index with equal weight of dimensions.

Model 2 uses the PCA-weighted business competition index.

* Significance at the 10% level

** Significance at the 5% level

*** Significance at the 1% level

**Table 8:** Regression of business competition (ϕ) on labor (Z).

Independent Variable	Dependent Variable: LnLabor	
	Model 1	Model 2
	Coefficient	Coefficient
Intercept	10.072***	9.646***
	(1.069)	(1.100)
Ln(*ϕ*)	-0.057	-0.068
	(0.045)	(0.042)
LnY	0.373***	0.411***
	(0.090)	(0.094)
Lnwage	-0.014**	-0.014**
	(0.007)	(0.007)
Dcov	-0.125	-0.100
	(0.084)	(0.079)
Dcov* Ln(*ϕ*)	0.090*	0.072
	(0.054)	(0.052)
R^2^	0.75	0.75
Wald-statistics	35.52***	35.91***

Source: authors’ calculation

Note: Standard errors in parentheses

Model 1 uses the business competition index with equal weight.

Model 2 uses the PCA-weighted business competition index.

* Significance at the 10% level

** Significance at the 5% level

*** Significance at the 1% level

**Table 9:** Regression of business competition (*ϕ*) on wage.

Independent Variable	Dependent Variable: LnWage	
	Model 1	Model 2
	Coefficient	Coefficient
Intercept	-8.775***	-8.432***
	(1.178)	(1.160)
Ln(*ϕ*)	0.991*	0.700
	(0.594)	(0.561)
LnY	0.298**	0.309**
	(0.123)	(0.125)
LnLabor	-0.200	-0.201
	(0.135)	(0.138)
R^2^	0.22	0.20
Wald-statistics	14.34***	12.69***

Source: authors’ calculation

Note: Standard errors in parentheses

Model 1 uses the business competition index with equal weight.

Model 2 uses the PCA-weighted business competition index.

* Significance at the 10% level

** Significance at the 5% level

*** Significance at the 1% level

**Table 10:** Regression of business competition (ϕ) on Human Development Index (HDI) (Z).

Independent Variable	Dependent Variable: LnHDI	
	Model 1	Model 2
	Coefficient	Coefficient
Intercept	3.785***	3.769***
	(0.135)	(0.135)
Ln(*ϕ*)	0.010	0.017**
	(0.007)	(0.007)
LnY/L	0.086***	0.083***
	(0.012)	(0.013)
Ln(S_h_)	0.021***	0.020***
	(0.008)	(0.007)
Ln(S_k_)	0.038*	0.040*
	(0.022)	(0.022)
Dcov	-0.003	-5.95*10^−5^
	(0.020)	(0.022)
Dcov* Ln(*ϕ*)	0.003	0.001
	(0.013)	(0.014)
R^2^	0.200	0.210
Wald-statistics	163.130	223.390

Source: authors’ calculation

Note: Standard errors in parentheses

Model 1 uses the business competition index with equal weight.

Model 2 uses the PCA-weighted business competition index.

* Significance at the 10% level

** Significance at the 5% level

*** Significance at the 1% level

#### 4.2.1. Single equation model between business competition and Gross Regional Domestic Product (GRDP

As explained in the section of Research Model, the single regression indicating the relationship between business competition and welfare (Eq (4)) is firstly estimated. Table 4 shows the regression results of the three regression models used to determine the impact of business competition on GRDP using the business competition index measure with the same weight for competition dimensions (All variables are transformed into natural logarithms to have a relationship in the growth meaning). Based on the availability of sectoral and provincial data, the estimated model allows for only the relationship between GRDP and business competition using annual data. Only GRDP data and business competition index data have a sectoral basis in each province for each year.

The first model estimated the effect of business competition on GRDP using combined pooled data for economic sector, province, and year. The first model’s estimation results show that GRDP was highly elastic to business competition. The coefficient of 5.635 indicated that if the business competition index increased by 1%, the GRDP increased by 5.635%, ceteris paribus. The business competition index significantly affected GRDP at the 1% critical level.

The second model in Table 4 is a model for estimating the effect of business competition on GRDP using pooled data combining province and year. The second model’s estimation results show that GRDP was highly elastic to business competition. The coefficient of 5.389 indicated that if the business competition index increased by 1%, the GRDP increased by 5.389 percent, ceteris paribus. The business competition index significantly influenced GRDP at the 1% critical level.

Moreover, the third model in Table 4 estimated the effect of business competition on GRDP growth using data pooling economic sector and year. The third model’s estimation results show that GRDP growth was also highly elastic to business competition. The coefficient of 17.520 indicated that if the business competition index increased by 1%, it increased the GRDP by 17.520%, ceteris paribus. The business competition index significantly influenced GRDP at the 1% critical level. This result also indicated that business competition in the economic sectors tended to have a greater impact on the economic growth.

Table 5 also shows how business competition affects economic growth by using the measure of the business competition index with PCA weights for each of its constituent dimensions. In general, business competition had a positive effect on economic growth (LnY), with the 1% critical level. The elasticity of GRDP on business competition was also very elastic, as found in the model using the business competition index measurement, which uses the same weight for all dimensions of business competition. The elasticity of GRDP growth on business competition was above 1 for the three models: 5.446; 5.021; and 14.648 for models 1, 2, and 3, respectively.

The robust results of the effects of business competition on the economic growth in the Tabel 4 and Tabel 5 supported the findings from [8, 20–22] who found that competition affected significantly the economic growth. This study also confirmed that competition can be a driver for a competitiveness of the regional and national economy (see [51]).

#### 4.2.2. Model of business competition and regional productivity

Table 6 shows the effect of the business competition index on welfare (Eq (1)) using models (1) and (2) with pooled data combining province and year. The results of the model estimation show that there was a positive effect of business competition on regional productivity in Indonesia. Using business competition indicators based on equal weights and PCA weights, each business competition significantly increased regional productivity at the 10% and 1% critical levels, respectively. The results support the research from [18, 23–26, 28] who found a positive relationship between competition and productivity. These findings suggest that competition could improve regional productivity and was expected to have a positive impact on regional welfare. Furthermore, the positive effect of competition on the productivity of all sectors can improve the economic performance of the regions, which can increase the regional budget allocation from the state authorities for the regions and improve the welfare in the regions even more.

During period 2018–2020, education and investment spending did not accelerate economic growth and tended to lead to a decline in economic growth, as shown in the Table 6. This is presumably because the costs of education and investment expenditures have an impact in the long term, so in the short term during the 2018–2020 period, education and investment expenditures still have a negative effect on regional economic growth. Moreover, the condition of economic growth did not differ significantly between before and during the COVID-19 pandemic, although there were indications of a decline in economic growth seen from the COVID-19 pandemic dummy coefficient (Dcov). COVID-19 pandemic dummy variable had negative coefficients for both models with the business competition index using the same weight and PCA weight.

#### 4.2.3. Model of business competition and productivity growth

Furthermore, Table 7 shows the results of an estimation of the effect of business competition on economic growth as measured by growth in labor productivity (Eq (2)). The estimation results of the model show that there was a positive effect of business competition on growth of GRDP per worker at the 5% critical level when using business competition indicators with PCA weights. Every 1% increase in the business competition index increased productivity growth by 0.136%, ceteris paribus. Moreover, the population had a negative effect on productivity growth at the 1% and 5% critical levels, respectively, for both models with equal weight and the PCA weight. The results supported the finding of [27] who suggested that the competitive pressures can lead to more efficient market and innovation. These benefits could improve the productivity growth.

#### 4.2.4. Business competition model and other welfare indicators

Tables 8–10 shows the estimated model of Eq (3) relating business competition and other measures of welfare. Based on Table 8 it can be seen that business competition had no significant effect on labor in both models using business competition index with same weight and PCA weight. GRDP had a positive influence on the demand for labor at 1% critical level in each model with the business competition index measure using the same weight and PCA. Business competition tended to have no effect on labor. This is possible when market reforms are not carried out as a whole, as found in the research of [29], where business competition could improve the performance of inputs, including labor inputs, when market reforms are carried out as a whole.

From Table 8, it can also be seen that the COVID-19 pandemic (Dcov) also did not cause a significant decrease in the amount of employment at the 10% critical level for each model with a business competition index measure using equal weights and PCA. Wage (Lnwage) caused a decrease in the number of demands for labor at 5% critical level for each model using business competition indicators using equal weights and PCA weights. The results were relevant, since the higher price of labor may lead to higher cost for firms causing the decline of the labor demand.

Moreover, business competition increased the demand for labor during the COVID-19 pandemic (Dcov* Ln(*ϕ*)) at the 10% critical level only in a model using the business competition index with the same weight of dimensions. Although the previous research never investigated the relationship between the two variables in the pandemic Covid-19, the nature effect of competition on the labor demand may not change. This is also in line with the result of the insignificant effect of the pandemic Covid-19 on the employment in this research. Thus, this research still supported the research from [28–33] who found that the higher competition could support the higher demand for labor.

Table 9 shows the effects of business competition and other factors on the wage level of workers. Business competition had a positive and significant effect on labor wage at the 10% critical level in the model using the business competition index with the same weight measure. It indicates that business competition encourages labor productivity and can also promote higher wages, as also found by [34]. The competition may increase the wage through the nominal wage (because of higher productivity) or real wage (because of the lower product price).

Table 9 presents that GRDP positively affected wage at the 5% critical level in each model using the business competition index with the same weight and PCA measure. Regional economic performance can encourage higher wages through higher output which may increase labor demand or productivity. In addition, using the same weight and PCA, the absorption of labor had no significant effect on wages. With respect to that, the higher wage may be caused by the productivity improvement, instead of labor demand. This also indicates that wage can increase from the basis of the minimum wage because of the productivity.

Table 10 provides an estimation model of the factors that influence the human development index (HDI). From Table 10, it can be seen that business competition affected the human development index only in the model with a business competition index using PCA weight at the 5% critical level. Every 1% increase in the business competition index will increase the HDI by 0.017%, ceteris paribus. Regional productivity also had a positive effect on HDI at the 1% critical level in each model using the business competition index with the same weight as well as the PCA weight. Education expenditure also had a positive effect on HDI at the 1% critical level in each model using the business competition index with equal and PCA weights. Moreover, investment had a positive effect on HDI at the 10% critical level in each model using the business competition index with the same weight and PCA weight. The positive effect of competition on the HDI was in line with research of [40–43] who found that competition would benefit the consumers in terms of improvement in the income, quality of health and the quality of education.

## 5. Conclusions and policy implications

The purpose of this study is to investigate the effect of business competition on welfare in Indonesia. This research found that regions that had high welfare tend to have a high level of business competition. Over the past three years, the provinces that include Jakarta, Central Java, East Java and West Java were the ones with the highest business competition index, with an average score index above 5. Meanwhile, West Papua, Papua, Gorontalo, West Sulawesi, and Lampung had a low business competition index (score less than 4.5) over the last three years. Furthermore, the sectors that had experienced the highest level of competition index in the previous three years included wholesale and retail trade, auto and motorcycle maintenance; financial services, insurance; educational services; information and communication; and business services. Furthermore, the sectors that had experienced the lowest level of competition index in the previous three years included the mining and quarrying sector; water supply, waste management and recycling; and electricity and gas supply.

This research generally found a relationship between the business competition index and national welfare as measured by gross regional domestic product, regional productivity, productivity growth, wages, and the human development index. The labor absorption was not significantly affected by business competition.

This research has a limitation, since this research only applies three-year period of business competition and welfare data for the estimation. In spite of this, the level of competition index was consistent at the provincial and sectoral level data during the three-year period. Thus, this research suggests that the government and KPPU should continue to encourage the mainstreaming of business competition in all economic sectors and regions in Indonesia. The government can carry out comprehensive market reforms (such as lowering the barrier to entry) so as to encourage the strengthening of the impact of business competition on overall welfare measures, including other welfare indicators such as employment.

Furthermore, the government must encourage economic sectors so that potential new firms can easily enter the market causing higher competition in the market. Sectors of the mining and quarrying sector; water supply, waste management and recycling; and electricity and gas supply, which consistently stayed in the same lowest ranks during the period 2018–2020, should be revitalized or opened for the investment so that they can be widely provided by the private firms. Furthermore, provinces that are indicated to always have the lowest business competition index such as West Papua, Papua, Gorontalo, West Sulawesi, and Lampung, are encouraged to be more open to investors and new firms, with the possibility of providing incentives.

## Supporting information

S1 Table Research data. (XLSX)
